# Randomized clinical trial of a novel food insecurity intervention for patients with opioid use disorder: Unexpected improvements in depression, quality of life and pain outcomes

**DOI:** 10.1192/j.eurpsy.2026.10910

**Published:** 2026-07-31

**Authors:** S. Sigmon

**Affiliations:** Psychiatry, University of Vermont College of Medicine, Burlington, United States

## Abstract

**Introduction:**

The adverse consequences of illicit opioid use (e.g., overdose, infectious disease) have been the focus of intensive research efforts. However, other serious health problems among individuals with OUD have received far less attention. Food insecurity (FI) is 4-7 times greater among individuals with OUD than the general US population and associated with increased drug use, psychiatric distress, sexual and drug risk behaviors and a two-fold risk of premature death.

**Objectives:**

To evaluate the feasibility, acceptability and initial efficacy of a meal delivery intervention for reducing FI and other areas of psychosocial functioning among adults with OUD.

**Methods:**

Participants (n=50) who were enrolled in MOUD treatment and met criteria for FI (>3 on the US Household Food Security Survey (FSS)) were randomized to one of two 12-week conditions. **Nutritional Education (NE; n=25)** participants received brief NE, a list of FI-related community resources, and assistance contacting resources of interest. **NE+Meal Delivery (NE+MD; n=25)** participants received NE plus premade, refrigerated meals delivered weekly by a commercial service. Both groups completed monthly assessments of FI, depression, quality of life, and illicit drug use.

**Results:**

Participants were 39.2+7.3 years old, 58% female and 82% white. Participants’ mean baseline FSS score was 8.2+3.1. Following randomization, retention was similar between the groups (88% and 84% in NE+MD and NE conditions, respectively). NE+MD participants demonstrated significantly greater decreases from intake in FSS score vs. controls, with mean scores lower in NE+MD vs. controls at all three post-intake timepoints (p’s<.05). Among NE+MD participants, a total of 2,976 meals were successfully delivered and meal enjoyment and convenience were rated at 81 and 93, respectively (range:0-100).

Pilot data also suggested several potential associations between FI and other areas of functioning. NE+MD participants’ scores on the Beck Depression Index were significantly lower than intake at Study Week 4 and 8 (p’s<.05). No such changes in BDI were observed in NE participants. On the RAND-36 QoL Health Survey, NE+MD participants experienced significant improvements (p’s p<.05) on four subscales, with greater scores on the subscales of Mental Health, Role Emotional, Bodily Pain, and General Health. No changes were observed on any subscales for NE participants. Finally, while there were no significant differences between groups on illicit opioid abstinence during the study, NE+MD participants provided 23% more urine specimens testing negative for illicit opioids on assessment days at which they were FS vs. FI.

**Conclusions:**

Preliminary results suggest the initial feasibility, acceptability and efficacy of this intervention for improving FI and other areas of psychosocial functioning among individuals with OUD.

**Disclosure of Interest:**

None Declared

